# Outcome of the endoscopic repair of frontal sinus cerebrospinal fluid leak

**DOI:** 10.1016/j.amsu.2021.102887

**Published:** 2021-09-23

**Authors:** Wael F. Ismaiel, Mohamed H. Abdelazim, Ahmed Younes, Mahmoud E. Alsobky, Abdulkarim Hasan, Ahmed M. Taha

**Affiliations:** a -Department of Otorhinolaryngology, Faculty of Medicine, Al-Azhar University, New Damietta, Egypt; b -Department of Pathology, Faculty of Medicine, Al-Azhar University, Cairo, Egypt; c - Department of Neurosurgery, Faculty of Medicine, Al-Azhar University, New Damietta, Egypt

**Keywords:** CSF leak, Draf III, Endoscopic surgery, Frontal sinus

## Abstract

**Introduction:**

and Objectives: Leakage of cerebrospinal fluid (CSF) from the frontal sinus is a challenging condition facing the ENT surgeon. Repair of this condition has been changed nowadays due to the newer instruments and techniques of nasal endoscopy. This study aims to evaluate the outcome of frontal sinus CSF leak endoscopic repair.

**Patients and methods:**

Twenty-seven patients who had frontal sinus CSF leaks were included in this study. They were 9 females and 18 males. They underwent endoscopic repair of the leak site at the period of five years from 2015 to 2020. A retrospective evaluation of these patients includes reconstructive procedures, complications, and postoperative follow-up.

**Results:**

The frontal leaks were present in the frontal recess (8 patients, 29.6%), ethmoidal roof (5 patients, 18.5%), and the majority was in the posterior wall (14 patients, 51.9%); 11 in the medial side and 3 in the lateral side. All cases, 27 (100%) were treated successfully, no failed treatment was observed. Postoperative complications were minimal; two patients had elevated intracranial pressure (ICP), infection with fever were found in four patients (7.4%), and meningitis was observed in only two cases (7.4%), treated conservatively.

**Conclusion:**

For frontal sinus CSF leak repair, the endonasal endoscopic approach is the treatment of choice due to higher success rates and lower morbidity profile. A favorable result is possible with proper diagnosis, precise localization, and an appropriate strategy.

## Highlights

• CSF leakage from the frontal sinus is a challenging condition for ENT surgeons.
• Repair has been changed nowadays due to the newer techniques of nasal endoscopy.
• We report favorable results of the endonasal endoscopic approach in 27 patients.
• Proper diagnosis and precise localization are acheived.

## 1 Introduction

Leakage of cerebrospinal fluid from the frontal sinus is a challenging condition facing the ENT surgeon. Extracranial repair of frontal sinus CSF leaks has been attempted using osteoplastic flaps, obliteration, and cranialization, all of which have a significant incidence of morbidity [1].

Causes of frontal CSF leaks include trauma, iatrogenic leak, congenital malformations of the skull base, and malignancy [2,3].

Frontal CSF leaks that occur spontaneously often occur at regions of wall weakness that rupture under hydrostatic pressure caused by higher ICP, such as anterior cribriform plate, the ethmoid roof, and posterior wall [4].

Repair of this condition has been changed nowadays due to the newer instruments and techniques of nasal endoscopy. This condition must be managed carefully and predicts their incidence among other sites of nasal CSF leak [2,5].

Nowadays, the technique of choice is the endonasal endoscopic approach. However, the unique anatomical structure, the vital organs around, the ostium narrowing, and the acute nostril to the frontal sinus; all form the endoscopic approach technical complexity [6]. Draf described many techniques for frontal sinusotomies that ranged from anterior ethmoidectomy (Draf type I) to the extended Draf type III approach [7].

Though Draf III method provides good visibility of the frontal sinus, in 65% of cases, the defect can be only reached via the larger ostium, as recently highlighted [6]. The present study aims to assess the outcome of the endoscopic repair of the frontal CSF leaks in a tertiary care hospital.

## 2 Patients and Methods

This retrospective study was conducted on 27 patients with frontal sinus leaks. They were 18 males and nine females. It was performed in ENT departments of Al-Azhar University Hospital, New Damietta, Egypt, for a period of five years, from May 2015 to May 2020.

Written informed consent was given by the patients for their clinical records to be used in this study. The Institutional Review Board (IRB00012367-21-01-010) approval was obtained from Al-Azhar University, Faculty of medicine in Dameitta, for this study.

The inclusion criteria included: patients who underwent endoscopic frontal sinus repair. Traumatic or spontaneous patients with a CSF leak at a minimum of six months not responding to conservative measures were included.

Subjects were excluded if they had a known malignancy or patients with frontal leak due to extensive tumor resection or intracranial injury.

## 3 Methods

All patients in this study were evaluated during the follow-up period. Patients' age, sex, occupation, residence, and telephone number were recorded for each participant for demographic purposes. General and local examinations were also performed preoperatively as usual.

Medical history, surgical approach, leakage site, complications, reconstruction technique, and follow-up were recorded.

### 3.1 Radiologic evaluation

1.1. Simple Skull Xray: demonstrate fracture and pneumocephalus.

**Fig. 1:**
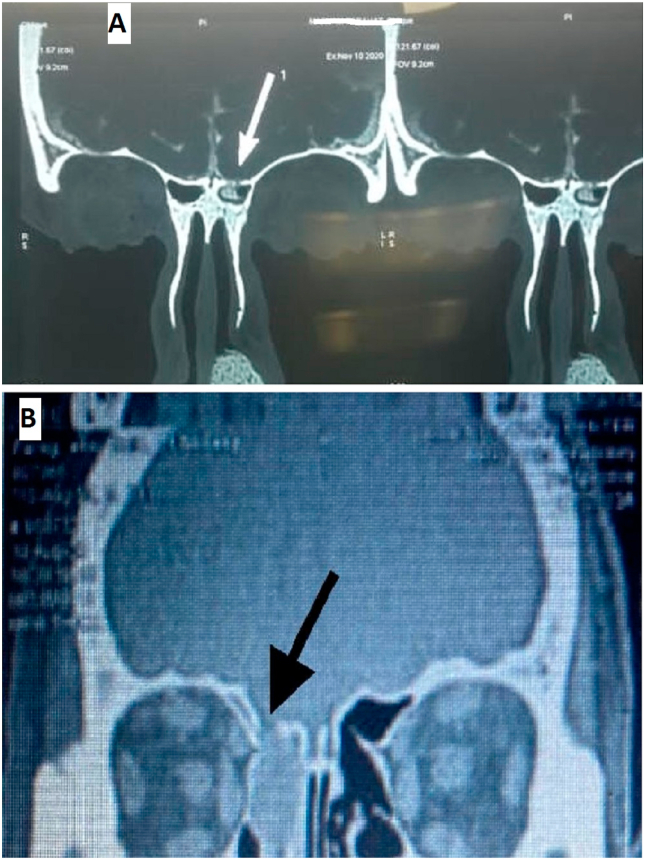
CT scan coronal cuts pictures; A) Nose and paranasal sinus showing frontal sinus recess defect. B) Nose and paranasal sinus showing a defect in the medial part of frontal sinus posterior table.

1.2. High-Resolution CT scans show the fracture location and accompanying meningocele (Fig. 1).

1.3. Magnetic resonance imaging (MRI) to detect fistula and tract size and length.

### 3.2 Laboratory tests

There is no need for additional confirmatory testing in case of identifying skull base fractures using CT and CSF leak. Beta-2 transferrin assay was preferred if a confirmatory test was required due to its excellent sensitivity and specificity. Beta-2 transferrin is almost exclusively detected in CSF. Transferrin beta-2 is not present in the blood, tears, or nasal secretions. Intrathecal fluorescein is typically utilized during the operation to verify and identify the CSF leak fistula. CSF cytology examination by the anatomical pathologist was performed for one patient to exclude metastasis of an old colorectal cancer and the CSF was negative for malignancy as well as the free MRI scan.

### 3.3 Surgical procedure

**Fig. 2:**
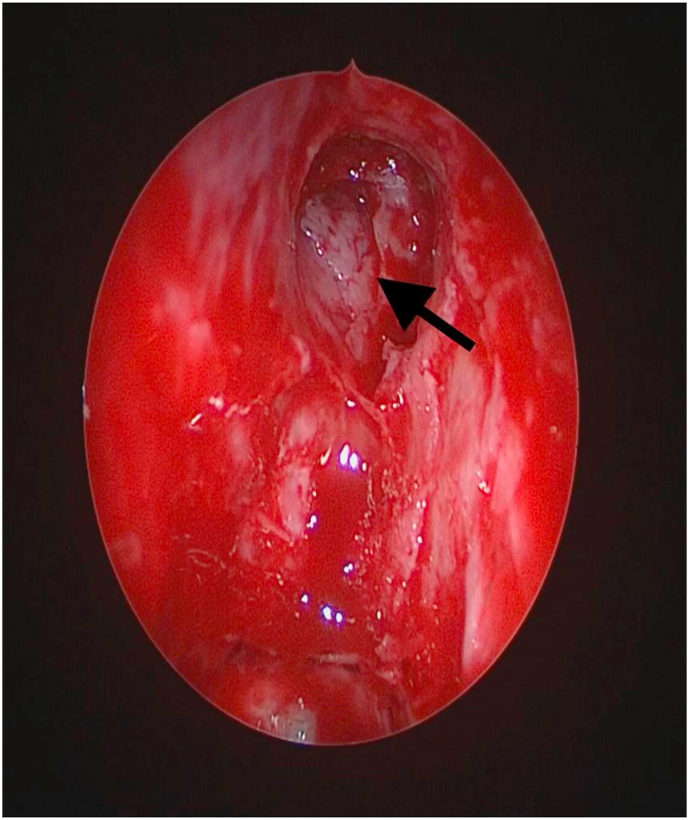
Endoscopic localization of a defect in the medial part of the posterior table of the frontal sinus.

**Fig. 3:**
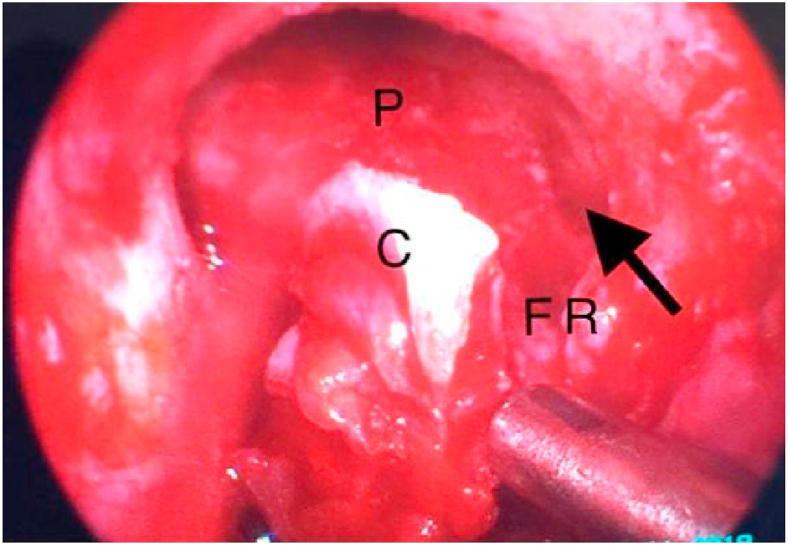
Endoscopic picture showing Draf III, C = cribriform palate of ethmoid, P = posterior table of the frontal sinus, FR = frontal recess with pointing arrow to the site of defect.

All cases were treated by endonasal endoscopy (Fig. 2, Fig. 3).

3.1. Complete sphenoethmoidectomy.

3.2. Draf type IIa, IIb, and III according to defect location.

3.2.1. Defect less than 3 mm closed by a plug of fat and facia lata or middle turbinate mucosa.

3.2.2. Defect more than 3 mm closed by underlying facia lata, underlying cartilage, and overlay facia lata.

### 3.4 Statistics

SPSS software (Chicago, Illinois) version 23 was used for data analysis. Descriptive measures (means, standard deviations, and frequencies) were calculated for all parameters. To determine P values using the Pearson's correlation test and a χ^2^ test and 95% confidence interval can be expressed in terms of a single sample. P < 0.05 was considered statistically significant.

We report the results of this study in accordance with the STROCCS reporting statements [8].

## 4 Results

The present study included 27 patients with frontal sinus leaks. They were 18 males and 9 females, they showed a statistically significant difference (P < 0.001). The patient's average age was 45.7 ± 12.4 years (range, 27–63 years). Most of patients were traumatic (63%), while spontaneous patients were (37%). The follow-up period ranged from 18 to 36 months with a mean of 24 ± 9.86 months, difference between lower and upper levels of confidence interval (CI) showed a statistically significant difference (P < 0.01). The frontal leak was spontaneous in 11 patients (40.7%) and traumatic in 16 patients (59.3%), of which 13 cases were accidental, and only three cases were iatrogenic. The frontal leaks were present in the frontal recess (8 patients, 29.6%), ethmoidal roof (5 patients, 18.5%), and the majority in the posterior wall (14 patients, 51.9%); eleven in the medial side and three in the lateral side (Table 1).

**Table 1:** Demographic data of the studied patients.

Frontal sinus leak patients			χ^2^	P value
Gender	**Number (27)**	**Percent (%)**		
• Males	18	66.7	19.34	0.000
• Females	9	33.3		
	**Mean ± SD**	**Range**	**95% CI**	**P value**
Age (years)	45.7 ± 12.4	27–63	2.36–8.21	0.001
Follow up period (months)	24.0 ± 9.86	18–36	0.94–5.37	0.002
Site of leak	**Number (27)**	**Percent (%)**	**95% CI**	**P value**
• Frontal recess	8	29.6	0.588–1.213	0.176
• Ethmoidal roof	5	18.5	0.437–1.109	0.248
• Posterior wall:	14	51.9	1.23–5.74	0.005
➢ Medial side	11			
➢ Lateral side	3			

χ^2^: Chi square, CI: Confidence interval, P < 0.01 = highly significant.

Regarding the surgical technique, Draf IIa was used in frontal recess and ethmoidal roof leak in 13 patients (48.2%). Draf IIb was used in the medially located part of the posterior wall in 11 patients (40.7%). Draf III was used in the lateral aspect of the posterior wall in only three patients (11.1%) (Table 2).

**Table 2:** Surgical technique and the site of the leak.

Surgical technique	Defect site	Frontal sinus leak patients	
		Number	Percent (%)
Draf IIa	Frontal recess and ethmoidal roof	13	48.2
Draf IIb	Posterior wall: medial part	11	40.7
Draf III	Posterior wall: lateral part	3	11.1
Total		27	100

All cases, 27 (100%), were treated successfully, no treatment failure was observed. Postoperative complications were minimal; two cases had elevated intracranial tension (ICT), infection and fever were found in four patients (7.4%), and meningitis was observed in only two cases (7.4%), treated conservatively (Table 3).

**Table 3:** Outcome of treatment of frontal sinus leak.

Outcome:	Number (27)	Percent (%)	χ^2^	P value
• Successful treatment	27	100	78.94	0.000
• Failed treatment	0	0.0		
**Complications:**			**95% CI**	**P value**
• Elevated intracranial tension	2	7.4	0.14–0.37	0.798
• Fever or infection	4	14.8	0.22–0.35	0.694
• Meningitis	2	7.4	0.14–0.37	0.798
• Recurrence or re-leak	0	0.0	N/A	N/A
• Anosmia	0	0.0	N/A	N/A

χ^2^: Chi square, CI: Confidence interval, P < 0.001 = highly significant.

## 5 Discussion

Frontal sinus leaks are a rare situation and their surgical management is difficult. In their systematic review, Psaltis et al. [9] found that the most commonly affected was ethmoid/cribriform plate, followed by sphenoid, then frontal sinus. Banks et al. [10] conducted a large case series on patients with CSF leaks treated over 21 years; only 11.4% of patients showed leaks from the frontal sinus.

Although the most common cause of CSF rhinorrhea has been considered trauma, Psaltis et al. systematic review, which included 1778 fistula repairs, stated a higher incidence of spontaneous CSF leaks that represented 40.7% of our series [9]. In the current study, the most common etiology was trauma (59.3%).

In agreement with the current study, Jahanshahi et al. [11] stated in their retrospective review that the most common etiology was trauma (18 of 24 patients), followed by spontaneous leaks (6/24) and without accounting for the defects caused by excision of the tumor. Gâta et al. [6], with 77.2% of CSF leaks caused by trauma and the rest being spontaneous, had similar findings to us.

Regarding the surgical technique, Draf IIa was used in frontal recess and ethmoidal roof leak in 13 patients (48.2%), Draf IIb was used in the medial part of the posterior wall in 11 patients (40.7%). Draf III was used in the lateral aspect of the posterior wall in only 3 (11.1%) of patients.

Frontal sinus trephination is an effective tool expanding the technique of skull base surgeries [12,13]. Trephination can be large enough to establish a surgical corridor for the endoscope and other surgical equipment, hence increasing sinus access. Trephination of the frontal sinuses is a relatively safe surgery with a low complications risk and great cosmetic outcomes [14].

Jones et al. [15] found, among their 24 cases, that frontal sinusotomy Draf IIb was considered the most common approach (21 patients). In the study by Jahanshahi et al. [11] on 24 patients with frontal sinus leak, the technique was similar, with Draf IIb being employed in 20 patients, Draf III in three patients, while Draf IIa in only one patient.

Woodworth et al. [16] performed the first endoscopic repair of frontal CSF leaks. Six patients were successfully treated in their study, and one patient required only an adjuvant osteoplastic flap without obliteration. Frontal CSF leaks were successfully treated in all cases (27 individuals) in our study. This was parallel to Gâta et al. [6], who successfully treated endoscopically over 95% of cases. It is possible to preserve a patent frontal sinus drainage channel while concurrently correcting skull base defects [1,17].

Jones et al. [15] reported a 91.9% success rate of closure following the initial attempt, increasing to 97.3% with later endoscopic revision. The research enrolled 37 patients who were followed for an average of 48 weeks during 3.5 years. The reason that other trials success rates were lower than ours (100%) might be due to tumor-induced leaks, whereas our series eliminated several leaks. Jahanshahi et al. [11] reported a 95.83% success rate for endoscopic repair in a retrospective series identical to ours. Their study enrolled 24 individuals with frontal CSF leaks, and one patient with multiple skull base fractures suffered meningitis ten months after a successful surgery. The 100% success rate found in the present study was achieved by similar studies [6,18,19]. Most frontal sinus CSF leaks (77.2%) were endoscopically treated [6].

Postoperative complications were minimal; two cases had elevated intracranial tension (ICT), infection and/or fever was found in four patients (7.4%), and meningitis was observed in only two cases (7.4%), treated conservatively.

Gâta et al. [6] reported revision surgery rates that were low and carried out for minor complications in two patients. There are multiple reported postoperative complications of such endoscopic surgery included cerebral edema (increased ICT), anosmia, frontal lobe deficits, frontal lobe traction, intracerebral hemorrhage, prolonged hospital stay in addition to mucocele formation [1,20,21].

This research has the same limitations as any retrospective review. The data were gathered through patient files, thorough notes, and surgical recordings. Furthermore, due to insufficient data, correlations between anteroposterior diameters, the size of each defect, and the incidence of meningoceles could not be calculated. These limits could be addressed by conducting more studies with large number of cases.

## 6 Conclusion

Frontal sinus CSF leak repair via endonasal endoscopic approaches is the treatment of choice in recent decades due to lower morbidity profile and better success rate. A favorable result is possible with proper diagnosis, precise localization, and an appropriate approach.

• Draf IIa is recommended in frontal recess and ethmoidal roof leak.
• Draf IIb is recommended in the medial part of the posterior wall leak.
• Draf III is recommended in the lateral part of the posterior wall leak.

## Conflicts of interest

The authors declare no conflict of interest.

## Sources of funding

This study did not receive any funding from governmental or private organizations.

## Ethical approval

Ethical approval was obtained from Al-Azhar Faculty of medicine Dameitta branch: IRB00012367-21-01-010.

## Consent

NA.

## Author statement

Study concept or design: WFS, MHA, MEA, AY, AH, AMT.

Data collection: WFS, MHA, MEA, AY, AMT.

Data interpretation: WFS, MHA, AH, AMT.

Literature review: WFS, MEA, AY, AH.

Data analysis: MHA.

Drafting of the paper: ALL.

Editing of the paper: ALL.

Manuscript revision: ALL.

## Registration of Research Studies

ClinicalTrials.gov Identifier: NCT05024643.

## Guarantor

Dr. Wael Ismaiel.

## Institution of the study

Al-Azhar University Hospitals in New Damietta, Egypt.

## Ethical approval

Ethical approval was obtained from the Institutional Review Board.

## Provenance and peer review

Not commissioned, externally peer-reviewed.
